# A Frequency-Based Proxy Score for Ultra-Processed Food Consumption Among Adolescents: Associations with Lifestyle Factors and Tinnitus

**DOI:** 10.3390/nu18162603

**Published:** 2026-08-10

**Authors:** Milena Tomanic, Srđan Milovanović, Olivera Stanojlović, Dušan Mladenović, Dragana Davidović, Milan Dobrić, Milena Vesković, Katarina Djurdjević, Šućro Madžgalj, Dimitrije Zdravković, Aleksandra Jovanović, Maja Lačković

**Affiliations:** 1 Faculty of Medicine, Institute of Hygiene and Medical Ecology, University of Belgrade, 11000 Belgrade, Serbia; dragana.davidovic@med.bg.ac.rs (D.D.); katarina.djurdjevic@med.bg.ac.rs (K.D.); 2 Clinic for Psychiatry, University Clinical Centre of Serbia, 11000 Belgrade, Serbia; srdjan.milovanovic@med.bg.ac.rs (S.M.); maja.lackovic@med.bg.ac.rs (M.L.); 3 Faculty of Medicine, University of Belgrade, 11000 Belgrade, Serbia; milan.dobric@med.bg.ac.rs (M.D.);; 4 Faculty of Medicine, Institute of Medical Physiology, University of Belgrade, 11000 Belgrade, Serbia; 5 Faculty of Medicine, Institute of Pathophysiology, University of Belgrade, 11000 Belgrade, Serbia; dusan.mladenovic@med.bg.ac.rs (D.M.); milena.veskovic@med.bg.ac.rs (M.V.); 6 Cardiology Clinic, Institute for Cardiovascular Diseases “Dedinje”, 11040 Belgrade, Serbia; 7 Clinic for Mental Disorders “Dr Laza Lazarevic”, 11000 Belgrade, Serbia

**Keywords:** ultra-processed food, adolescents, dietary patterns, energy drinks, tinnitus, sleep

## Abstract

**Background/Objectives:** Ultra-processed food (UPF) consumption is increasingly recognized as part of broader adverse behavioral and health-related patterns in young people, yet evidence integrating dietary, sleep-related, behavioral, and health characteristics within the same adolescent population remains limited. The relationship between UPF-related dietary patterns and tinnitus is particularly understudied. This study examined factors associated with the highest category of a frequency-based proxy UPF score among adolescents in Belgrade, Serbia. **Methods:** A school-based cross-sectional study included 1285 students aged 15–19 years from 12 four-year secondary schools selected using a stratified multistage cluster probability sampling design. A frequency-based proxy UPF score was constructed from seven questionnaire-based food categories broadly consistent with UPF-related consumption patterns in the Serbian context. Participants were grouped into three categories using empirical tertile cut-points, and the highest category was modeled as the binary outcome in univariable and multivariable logistic regression analyses. **Results:** Lower academic achievement (aOR = 2.10; 95% CI: 1.38–3.21), alcohol consumption (aOR = 1.34; 95% CI: 1.04–1.74), and waking tired compared with waking rested (aOR = 1.58; 95% CI: 1.06–2.35) were associated with higher odds of being in the highest proxy-score category after adjustment for the measured covariates. Energy drink consumption frequency was also associated with the outcome after adjustment for the measured covariates (overall *p* < 0.001). Constant tinnitus showed one of the strongest adjusted associations (aOR = 2.55; 95% CI: 1.77–3.67). **Conclusions:** Adolescents in the highest category of the frequency-based proxy UPF score exhibited a broader pattern of academic, sleep-related, behavioral, and health characteristics, including a particularly strong association with constant tinnitus. These cross-sectional findings warrant longitudinal research to clarify temporal relationships and underlying determinants.

## 1. Introduction

Over recent decades, global food systems and dietary patterns have undergone substantial transformation, accompanied by a marked expansion in the availability, distribution, and consumption of ultra-processed foods (UPFs). Within the NOVA classification, foods are categorized according to the extent and purpose of industrial processing, with UPFs comprising industrial formulations typically manufactured from substances derived from foods and frequently containing additives used to modify flavor, color, texture, stability, or other sensory and technological properties. Common examples include carbonated soft drinks, packaged sweet and savory snacks, confectionery, mass-produced packaged baked products, reconstituted meat products, and ready-to-eat or ready-to-heat formulations [1]. The increasing penetration of these products into national food systems has been documented across regions and income levels, reflecting a broader global nutrition transition toward more highly processed diets [2].

Growing exposure to UPFs has become a major public-health concern because greater consumption has been associated with a broad range of adverse health outcomes. A recent umbrella review of epidemiological meta-analyses reported associations between higher UPF exposure and multiple adverse outcomes, with particularly consistent evidence across cardiometabolic, common mental disorder, and mortality domains [3]. These concerns are especially relevant in younger populations. Systematic reviews in children and adolescents have linked greater UPF consumption with excess body weight, greater adiposity, and a range of cardiometabolic abnormalities, although the strength and consistency of associations vary according to study design, exposure assessment, and population characteristics [4,5]. Adolescence may represent a particularly sensitive period because increasing autonomy over food choice coincides with rapid physical and neurobehavioral development and with the consolidation of dietary and lifestyle patterns that may persist into adulthood.

Importantly, high UPF consumption may not occur as an isolated dietary behavior. Emerging evidence suggests that it can coexist with a broader constellation of sleep-related, behavioral, educational, and substance-related characteristics. A systematic review and meta-analysis found that higher UPF consumption was associated with adverse sleep-related outcomes, with notable associations also observed among children and adolescents [6]. Recent adolescent research has reported an inverse association between UPF consumption and academic performance [7], while a nationwide population-based study found that greater UPF consumption was associated with alcohol use, tobacco smoking, and illicit drug use among adolescents [8]. These findings support the possibility that high UPF consumption may be embedded within a wider pattern of health-related behaviors rather than representing a discrete nutritional exposure.

Energy drink consumption is particularly relevant within this broader behavioral context. Although energy drinks constitute a heterogeneous product category and were considered separately from the frequency-based proxy UPF score in the present study, their use among young people has repeatedly been associated with adverse sleep characteristics and other health-risk behaviors. Systematic review evidence links energy drink consumption in children and young people with short sleep duration, poor sleep quality, alcohol use, smoking, other substance-related behaviors, and broader risk-taking patterns [9]. In our previous analysis of adolescents from Belgrade, regular energy drink consumption was negatively associated with sufficient sleep duration in both sexes [10]. Taken together, these findings raise the possibility that stimulant use, sleep-related characteristics, and highly processed dietary patterns may cluster within interconnected behavioral profiles during adolescence.

Nevertheless, the existing literature remains distributed across largely separate research domains. Studies commonly examine UPF consumption in relation to individual outcomes such as adiposity, sleep, mental health, academic performance, or substance use; whereas, fewer investigations simultaneously consider a broad range of sociodemographic, sleep-related, behavioral, and health characteristics within the same adolescent population. Such an integrated approach may be particularly informative because observed associations may reflect overlapping social environments, shared behavioral-regulation processes, reward-related tendencies, daily routines, or early metabolic vulnerability rather than independent and unrelated exposures. The available pediatric and adolescent literature therefore supports examining UPF consumption within a broader multidimensional health context while also underscoring the need for population-specific research [5,6,8].

Tinnitus represents an additional and comparatively underexplored dimension of adolescent health in relation to dietary patterns. Tinnitus is not confined to adulthood and is also reported among children and adolescents. A systematic review and meta-analysis estimated a pooled prevalence of approximately 13% for any tinnitus among children aged 5–17 years, while emphasizing substantial heterogeneity arising from differences in definitions and assessment methods [11]. Contemporary pediatric tinnitus literature has focused predominantly on prevalence, risk factors, hearing status, clinical assessment, and management, with major uncertainties remaining because of heterogeneous definitions and limited pediatric-specific evidence [12]. Evidence concerning dietary correlates of tinnitus in adolescence is particularly sparse, and the relationship between a broader UPF-related dietary pattern and constant tinnitus appears not to have been specifically characterized in adolescent populations.

Our previous work provided initial evidence that dietary characteristics may be relevant to constant tinnitus in adolescence. In the same underlying study population, greater consumption of fresh fruit and vegetables was inversely related to tinnitus frequency; whereas, higher consumption of sweetened carbonated beverages, fast food, and white bread was associated with greater odds of tinnitus [13]. These findings suggested that auditory symptoms in adolescents may warrant consideration within a broader dietary context rather than solely in relation to acoustic exposure. However, that earlier study examined individual food groups and did not evaluate whether constant tinnitus was associated with a broader UPF-related consumption pattern. The extent to which a broader UPF-related dietary pattern co-occurs with constant tinnitus in adolescents therefore remains largely unknown.

This question is relevant because a broader UPF-related dietary pattern may capture dimensions of dietary exposure that are not adequately represented by individual foods considered separately. UPF-rich dietary patterns may combine frequent exposure to energy-dense, highly palatable products with displacement of less processed, fiber- and micronutrient-rich foods and have been associated with a wide range of adverse cardiometabolic and other health outcomes [3,4,5]. Although these observations do not establish a mechanistic link with tinnitus, they provide a rationale for investigating whether constant tinnitus co-occurs with a broader UPF-related consumption pattern in adolescents. Such an approach is particularly pertinent given the limited evidence concerning potentially modifiable, non-acoustic correlates of tinnitus in younger populations [12].

Therefore, the aim of the present study was to examine associations between a frequency-based proxy UPF score and a broad range of sociodemographic, sleep-related, behavioral, and health-related characteristics among adolescents in Belgrade, Serbia. Specifically, we investigated patterns related to sex, school grade, academic achievement, parental education, sleep duration and subjective feeling after nighttime sleep, physical activity, alcohol consumption, smoking, energy drink consumption, sedative medication use, gambling participation, and constant tinnitus. We further sought to identify factors associated with the odds of being in the highest category of this score after multivariable adjustment. Particular attention was given to sleep-related characteristics, energy drink consumption, and constant tinnitus because these domains may provide insight into whether adolescents in the highest category of the proxy UPF score exhibit a broader pattern of behavioral and health-related characteristics.

## 2. Materials and Methods

### 2.1. Study Design and Participants

A school-based cross-sectional study was conducted among adolescents attending four-year secondary schools in Belgrade as part of a broader research project examining dietary habits, health-related behaviors, and lifestyle factors in the adolescent population. Data were collected during the 2019/2020 school year. The present study represents a secondary analysis of previously collected data. Selected analyses from the broader research project have been reported previously [10,13]. A detailed comparison of the previous and present analyses, including the study population, research questions, dietary variables, role of tinnitus, analytical approach, and incremental contribution of the present study, is provided in Supplementary Table S3.

The target population comprised students attending four-year secondary schools in Belgrade. The sample was selected using a stratified multistage cluster probability sampling design. In the first stage, stratification was performed by Belgrade municipality using available statistical data on the geographical distribution of four-year secondary schools. The number of schools selected from each stratum was determined in proportion to the total number of four-year secondary schools in the corresponding municipality, with the aim of reflecting their territorial distribution across Belgrade. Within each stratum, schools were then selected at random using a publicly available web-based randomization tool.

In the next stage, the planned number of participants was allocated across the selected schools taking into account the total number of students enrolled in each school. Balanced representation of all four grades of secondary education was also ensured, with approximately equal numbers of participants from each school year. Within each grade, classes were selected at random, and all students in the selected classes were invited to participate.

The original sampling plan targeted 5100 students from 17 public four-year secondary schools in Belgrade. Recruitment, collection of written consent forms, and questionnaire administration were implemented sequentially across schools. By January 2020, approximately 2500 signed consent forms had been collected across 12 schools, and questionnaire administration had begun. Fieldwork was interrupted in March 2020 because of COVID-19-related school closures and the transition to online teaching, while schools were at different stages of recruitment and data collection. Consequently, the available figures represented an interim fieldwork status rather than a completed recruitment process, and a conventional response rate could not be calculated using a single valid denominator. Because participant-level data were not collected from the remaining five selected schools, student profiles could not be compared directly between the schools included and not included before the interruption. Consequently, selection related to the interrupted recruitment process cannot be excluded. For the present study, additional data cleaning and consistency checks were performed to meet the requirements of the current analysis, yielding a final analytic sample of 1285 participants from 12 schools. Participation was voluntary and anonymous.

The study was conducted in accordance with the principles of the Declaration of Helsinki and was approved by the Ethics Committee of the Faculty of Medicine, University of Belgrade (approval No. 1550/XI-38). Written informed consent was obtained from participants aged 18 years or older. For participants younger than 18 years, written informed assent was obtained from the participant together with written informed consent from a parent or legal guardian.

### 2.2. Data Collection

Data were collected using an anonymous self-administered questionnaire completed by students during regular school hours in the presence of members of the research team. The administration procedure was standardized across all participating schools to ensure comparable conditions for data collection.

The questionnaire covered a broad range of characteristics relevant to adolescent dietary habits, health-related behaviors, and health indicators. Data were collected on sociodemographic characteristics, including sex, age, school grade, and parental educational level; dietary habits and frequency of consumption of selected food groups; sleep characteristics, including nighttime sleep duration and subjective feeling upon waking; and other health-related behaviors, including physical activity, alcohol consumption, smoking, frequency of energy drink consumption, and use of sedative medications. Age at the time of the survey was derived from the reported year of birth and categorized as ≤15, 16, 17, or ≥18 years. Maternal and paternal education were recorded separately and dichotomized as lower (primary or secondary school education) and higher (postsecondary education, including higher school, college, or university education); these categories were labeled lower and higher parental education, respectively. Additional information was collected on academic achievement and participation in different forms of gambling. Selected components of the questionnaire and related variables have been described in previous analyses from the broader research project [10,13]. Anthropometric measurements, including body weight and height, were planned for a subsequent phase of school-based data collection. This phase could not be completed because in-person fieldwork was discontinued following the COVID-19-related school closures; consequently, BMI and weight-status categories were unavailable for the present analyses.

Tinnitus was assessed using a standardized screening instrument. Details on its administration and the operational definition of constant tinnitus are provided in a separate subsection.

### 2.3. Dietary Assessment and Construction of a Frequency-Based Proxy UPF Score

Dietary habits were assessed using a food frequency questionnaire developed following the basic framework of the Youth/Adolescent Questionnaire (YAQ), a validated FFQ for older children and adolescents [14], and adapted by the research team to reflect locally relevant foods and eating practices [10].

As the dietary questionnaire was not originally designed to quantify ultra-processed food intake according to the NOVA classification, a frequency-based proxy UPF score was constructed for the present analysis. Seven questionnaire items were selected as indicators of the reported frequency of consumption of food categories commonly represented in the Serbian market by commercially prepared, packaged, or industrially formulated products. Most of the selected categories were broadly consistent with NOVA group 4; however, classification may vary according to product formulation, particularly for margarine, which may fall within NOVA group 2 or group 4 [1]. The selection was informed by a review of representative product labels available on the Serbian market. Because the questionnaire did not capture the specific brand, ingredient composition, or preparation method of the product consumed by each participant, the resulting composite measure was retained as a frequency-based proxy UPF score rather than interpreted as a definitive individual-level NOVA classification. The score comprised seven product categories: sugar-sweetened carbonated beverages, fast food, industrially produced pastries and baked goods, French fries, sweet and savory snacks, ketchup and mayonnaise, and margarine. The questionnaire item underlying the industrially produced pastries and baked goods component referred broadly to croissants, savory pastries, burek, puff-pastry products, and similar items. Because brand, formulation, place of preparation, and nutrient composition were not recorded, this component was interpreted as a heterogeneous proxy category rather than as a nutritionally or processing-defined group. The score was intended as an exploratory, frequency-based indicator of relative exposure to selected UPF-related food categories within this study population; it was not designed or validated to quantify total NOVA-defined UPF intake, portion size, or the proportion of dietary energy derived from UPFs. Because the questionnaire did not collect sufficiently detailed information on portion sizes or quantities consumed, total dietary energy intake could not be estimated and was therefore unavailable for covariate adjustment.

Energy-drink consumption was not included in the construction of the proxy UPF score and was analyzed separately as a lifestyle variable. Thus, any observed association was not generated by mathematical item overlap with the score; the potential conceptual proximity between the two measures is addressed in the Discussion. The original response options varied slightly across food categories but assessed consumption frequency, generally ranging from never or less than once per month to daily or multiple times per day. For construction of the proxy score, these responses were harmonized into a three-level ordinal scale: 0 for monthly or less often, 1 for weekly consumption, and 2 for daily or more frequent consumption. The primary frequency-based proxy UPF score was calculated as the sum of available component item values across the seven categories. The theoretical full-score range was 0 to 14 points, with higher scores indicating more frequent consumption of the selected food categories.

Based on the distribution of the total frequency-based proxy UPF score, participants were grouped into three categories using empirically derived cut-points approximating tertiles: lowest category, 0–3 points; middle category, 4–5 points; and highest category, ≥6 points. Unequal group sizes reflected tied values in the discrete score distribution.

For brevity, the frequency-based proxy UPF score is hereafter referred to as the proxy UPF score, and its three empirically derived categories are referred to as the lowest, middle, and highest score categories.

For binary logistic regression analyses, the proxy UPF score was dichotomized, with participants in the highest score category forming the outcome group and those in the lowest and middle score categories combined to form the reference group. The robustness of the findings to the choice of the primary threshold (≥6 points) was examined by repeating the multivariable model using alternative cut-offs of ≥5 and ≥7 points, while retaining the same covariates, reference categories, and complete-case approach. An additional sensitivity analysis evaluated the influence of margarine by reconstructing the frequency-based proxy UPF score using the remaining six component items. The six-item score was categorized according to its empirical tertile cut-points (0–3, 4–5, and ≥6 points), and the highest category was compared with the combined lowest and middle categories. The multivariable model specification, covariates, reference categories, and complete-case approach were identical to those used in the primary analysis.

### 2.4. Assessment of Lifestyle Factors and Health-Related Behaviors

Lifestyle factors and health-related behaviors were assessed using data obtained from the anonymous self-administered questionnaire. The analyses included sleep characteristics, physical activity, energy drink consumption, alcohol consumption, smoking, use of sedative medications, participation in different forms of gambling, and academic achievement.

Sleep characteristics were assessed based on self-reported average nighttime sleep duration and subjective feeling upon waking. The original sleep-duration response categories were ≤5 h, approximately 6 h, approximately 7 h, approximately 8 h, and ≥9 h per night. For the present analyses, these categories were recoded into ≤6 h, approximately 7 h, and ≥8 h. Subjective feeling after nighttime sleep was categorized as tired, variable, or rested. The tired category combined participants reporting feeling very tired or tired upon waking; whereas, the rested category combined those reporting feeling rested or fully rested.

Energy drink consumption was assessed using a detailed frequency-and-quantity item, with response options ranging from never or less than once per month to three or more cans per day (one can = 330 mL). For the present analyses, responses were recoded as monthly or less often, weekly, or daily consumption. Alcohol consumption was analyzed as a binary yes/no variable based on the question “Do you drink alcohol?”, with yes/no response options. Additional questionnaire items assessed beverage type, frequency, and quantity but were not used to define the binary variable included in the present analyses. Smoking and sedative medication use were assessed using the questions “Do you smoke?” and “Do you take tranquilizers?”, respectively, both with yes/no response options. Participation in gambling activities was assessed according to the reported type of activity and classified as no gambling participation, chance-based games, sports betting, or higher-risk gambling activities. For the present analyses, usual weekly physical activity was categorized as none, once or twice weekly, or three or more times weekly. These measures were intended to capture usual or current behavior at the time of the survey rather than lifetime exposure; no fixed look-back interval, such as the previous 30 days or 12 months, was specified. The assessment and recoding of energy drink consumption and sleep duration in this study population have been described previously [10]. Academic achievement was assessed based on self-reported performance during the previous school year. In the Serbian secondary-school grading system, individual subject grades range from 1 (insufficient/failing) to 5 (excellent), while the annual grade-point average ranges from 2.00 to 5.00 among students who successfully complete the school year, with 5.00 representing the highest possible achievement. The original response categories were 2.00–2.99, 3.00–3.99, 4.00–4.99, and 5.00. For the present analyses, participants were classified as having lower academic achievement (2.00–3.99), higher academic achievement (4.00–4.99), or outstanding academic achievement (5.00).

### 2.5. Assessment of Constant Tinnitus

Tinnitus was assessed using the six-item version of the Tinnitus Screener, developed to distinguish tinnitus from transient auditory sensations and to classify tinnitus according to its temporal pattern [15]. A Serbian-language version was used in the present study, with no changes made to the content of the individual items.

For the purposes of the present analysis, constant tinnitus was operationalized as a binary health-related variable based on the Tinnitus Screener item assessing whether tinnitus was perceived in a quiet environment. Participants selecting “always” were classified as having constant tinnitus; whereas, those selecting either of the other non-missing response options were classified as not having constant tinnitus. In the earlier tinnitus-focused publication based on the same underlying survey [13], completeness was determined at the level of the full six-item Tinnitus Screener; whereas, the present analysis derived the constant-tinnitus variable from the original item-level responses. Participants with a valid response to the item assessing tinnitus in a quiet environment could therefore be classified even when another Tinnitus Screener item was incomplete. The response to the classification item was initially missing for 31 participants. Review of the remaining item-level responses showed that 23 of these participants had negative responses to all other Tinnitus Screener items and were therefore classified as not having constant tinnitus; whereas, the remaining eight participants could not be classified and were retained as missing. This reconstruction yielded 1277 valid classifications, while eight participants remained unclassified. Record-level verification additionally identified one coding inconsistency: one participant who met the criterion for constant tinnitus had been coded as not having constant tinnitus and was recoded accordingly, resulting in 165 cases. The same instrument and substantive definition of constant tinnitus were retained across the two analyses.

### 2.6. Statistical Analysis

Statistical analyses were performed using IBM SPSS Statistics for Windows, Version 22.0 (IBM Corp., Armonk, NY, USA). Categorical variables are presented as frequencies and percentages, and differences across the three proxy-score categories were assessed using Pearson’s chi-square test. Binary logistic regression was used to examine associations with the highest proxy-score category, with the combined lowest and middle categories as the reference. Univariable models provided crude odds ratios (ORs), while the multivariable model included sex, academic achievement, sleep duration, subjective feeling after a night’s sleep, alcohol consumption, energy-drink consumption frequency, physical activity, and constant tinnitus. Covariates for the primary multivariable model were selected a priori on conceptual grounds rather than on the basis of statistical significance in the univariable analyses. Smoking and gambling were treated as more distal exploratory risk behaviors and were therefore retained in the univariable analyses but not included in the primary adjusted model. Results are reported as ORs or adjusted ORs (aORs) with 95% confidence intervals (CIs). Overall performance of the primary multivariable model was evaluated using the likelihood-ratio chi-square test, Nagelkerke’s R^2^, and the Hosmer–Lemeshow goodness-of-fit test. Multicollinearity was assessed using tolerance and variance inflation factor (VIF) values derived from an auxiliary linear model containing the same indicator-coded predictors as the adjusted logistic regression model. As an additional sensitivity analysis, the final primary model, which included physical activity, was further expanded by adding grouped age and maternal and paternal educational levels as categorical covariates. Maternal and paternal educational levels were dichotomized as lower (primary or secondary school education) and higher (postsecondary education, including higher school, college, or university education). Descriptive and univariable analyses used available data; whereas, the final primary multivariable analysis used complete cases (n = 1204). A sensitivity analysis using a prorated version of the primary proxy score was performed to address missing responses across the seven component items. An approximate weekly equivalent score (0.25, 1, and 7 for monthly or less, weekly, and daily or more frequent consumption, respectively) was prorated for participants with at least six valid items, empirically categorized, and compared with the original 0–1–2 score in the same complete-case sample (n = 1191) using identical covariates. The original three-category proxy-score outcome was additionally examined using ordinal logistic regression with a logit link and the same covariates as the final primary model. The proportional-odds assumption was assessed using the test of parallel lines, and model fit was evaluated using the Pearson and deviance goodness-of-fit tests. Further sensitivity analyses examined alternative score thresholds, a six-item score excluding margarine, and two extreme-case classifications of missing constant-tinnitus responses. Participants included in and excluded from the complete-case model were compared by sex, academic achievement, and proxy-score category using Pearson’s chi-square test and Cramer’s V; full results are presented in Supplementary Table S6. School identifiers were not available in the de-identified analytical dataset; therefore, cluster-robust or multilevel estimation could not be performed. All tests were two-sided, with *p* < 0.05 used as a conventional threshold for statistical significance. No formal adjustment for multiple comparisons was applied; given the exploratory nature of the study and the number of comparisons across the descriptive, regression, and sensitivity analyses, the reported *p*-values should be interpreted as exploratory rather than confirmatory.

## 3. Results

### 3.1. Participant Characteristics Across Categories of the Frequency-Based Proxy UPF Score

The analytical sample comprised 1285 adolescents from 12 schools. Based on the grouped age variable, 309 participants (24.0%) were aged ≤15 years, 341 (26.5%) were aged 16 years, 239 (18.6%) were aged 17 years, and 396 (30.8%) were aged ≥18 years. The lowest, middle, and highest categories of the frequency-based proxy UPF score included 412 (32.1%), 388 (30.2%), and 485 (37.7%) participants, respectively. Missing data for the characteristics presented in Table 1 ranged from 0 to 37 participants (0–2.9%).

Of the 1285 participants, 1204 (93.7%) were included in the final primary complete-case multivariable model and 81 (6.3%) were excluded. Exclusion was more frequent among male than female participants (8.5% vs. 5.0%; χ^2^(1) = 6.417, *p* = 0.011; Cramer’s V = 0.071), but did not differ across proxy UPF score categories (6.8%, 5.9%, and 6.2% in the lowest, middle, and highest categories, respectively; χ^2^(2) = 0.273, *p* = 0.872; Cramer’s V = 0.015). Among participants with non-missing academic-achievement data, exclusion also did not differ across lower, higher, and outstanding achievement categories (6.2%, 4.8%, and 6.1%, respectively; χ^2^(2) = 1.164, *p* = 0.559; Cramer’s V = 0.030; n = 1273). Full results are presented in Supplementary Table S6.

Responses to the Tinnitus Screener item used to classify constant tinnitus were initially missing for 31 participants: none in the lowest proxy-score category, one in the middle category, and 30 in the highest category. Review of the remaining item-level responses allowed 23 of these participants to be classified as not having constant tinnitus, leaving eight participants unclassified. All eight remaining unclassified participants were in the highest proxy-score category. Following reconstruction and record-level verification, 1277 participants had valid classifications, of whom 165 (12.9%) met the criterion for constant tinnitus. The distributions of sex, academic achievement, maternal and paternal education, subjective feeling after sleep, alcohol consumption, smoking, energy drink consumption frequency, physical activity, gambling participation, and constant tinnitus differed significantly across categories (all *p* < 0.05). In general, participants in the highest score category more frequently had lower academic achievement, lower parental education, tiredness after sleep, alcohol consumption, smoking, frequent energy-drink consumption, less frequent physical activity, gambling participation, and constant tinnitus. School grade, reported nighttime sleep duration, and sedative medication use did not differ significantly across categories.

### 3.2. Univariable Logistic Regression Results

Crude associations with the highest category of the frequency-based proxy UPF score are presented in Table 2. Female participants had lower odds of being in the highest score category than male participants (OR = 0.662; 95% CI: 0.525–0.834). Academic achievement showed a graded association: compared with outstanding achievement, the odds were higher among adolescents with lower achievement (OR = 3.315; 95% CI: 2.253–4.877) and among those with higher, but not outstanding, achievement (OR = 1.724; 95% CI: 1.200–2.477).

Among the behavioral and health-related characteristics, the strongest crude associations were observed for weekly and daily energy drink consumption (OR = 3.395; 95% CI: 2.432–4.740 and OR = 3.576; 95% CI: 1.909–6.698, respectively) and constant tinnitus (OR = 2.792; 95% CI: 1.998–3.902). Feeling tired or having a variable feeling after sleep, alcohol consumption, smoking, sports betting, and higher-risk gambling activities were also associated with higher odds of being in the highest score category (all *p* < 0.05). Physical activity was also associated with the outcome overall (*p* = 0.020); compared with no weekly physical activity, activity three or more times weekly was associated with lower crude odds of membership in the highest proxy-score category (OR = 0.657, 95% CI: 0.443–0.973; *p* = 0.036); whereas, activity once or twice weekly was not statistically significant. Reported sleep duration, sedative medication use, and participation in chance-based games were not statistically significant.

### 3.3. Multivariable Logistic Regression Results

The results of the multivariable logistic regression analysis are presented in Table 3. After simultaneous adjustment, sex, academic achievement, alcohol consumption, energy-drink consumption frequency, and constant tinnitus were associated with the highest category of the frequency-based proxy UPF score; whereas, the overall associations with reported sleep duration, subjective feeling after a night’s sleep, and physical activity were not statistically significant. The final multivariable model was statistically significant (likelihood-ratio χ^2^(13) = 137.276, *p* < 0.001), with a Nagelkerke R^2^ of 0.147. The Hosmer–Lemeshow test was non-significant (χ^2^(8) = 6.171, *p* = 0.628), indicating no evidence of lack of fit according to this test. Multicollinearity diagnostics indicated no problematic collinearity among the predictors; the minimum tolerance was 0.423 and the maximum VIF was 2.365. Female participants had lower adjusted odds of being in the highest proxy-score category than male participants (aOR = 0.701; 95% CI: 0.538–0.914; *p* = 0.009). Compared with adolescents with outstanding academic achievement, those with lower achievement had more than twofold higher adjusted odds of being in the highest score category (aOR = 2.102; 95% CI: 1.378–3.207; *p* = 0.001); whereas, the estimate for higher, but not outstanding, achievement was not statistically significant. Adolescents reporting feeling tired after sleep had higher adjusted odds than those reporting feeling rested (aOR = 1.575; 95% CI: 1.055–2.349); whereas, the estimate for a variable feeling after sleep was borderline and did not reach conventional statistical significance (aOR = 1.440; 95% CI: 0.999–2.076; *p* = 0.051). Alcohol consumption was associated with a more modest increase in adjusted odds (aOR = 1.344; 95% CI: 1.039–1.738).

The largest adjusted estimates were observed for energy drink consumption and constant tinnitus. Compared with monthly or less frequent consumption, weekly and daily energy drink consumption were associated with higher adjusted odds of being in the highest score category (aOR = 2.770; 95% CI: 1.942–3.952 and aOR = 2.359; 95% CI: 1.180–4.715, respectively). Constant tinnitus was associated with approximately 2.5 times higher adjusted odds (aOR = 2.548; 95% CI: 1.768–3.672). The prorated frequency-based proxy UPF score was available for 1264 participants, of whom 1191 had complete data for all variables included in the repeated multivariable model. Classification into the highest category versus the combined lowest and middle categories was identical to the primary classification among all participants with an available prorated score. The direction and magnitude of the principal associations were preserved, with full numerical results presented in Supplementary Table S2. The overall association with subjective feeling after a night’s sleep was not statistically significant (*p* = 0.104), although waking tired remained associated with the outcome compared with waking rested. Physical activity was not associated with the outcome overall (*p* = 0.138). The principal findings were generally preserved when alternative score thresholds of ≥5 and ≥7 points were applied. Lower academic achievement, waking tired, weekly and daily energy-drink consumption, and constant tinnitus remained associated with the outcome under both alternative classifications. Physical activity was associated with the outcome only at the ≥5-point threshold (overall *p* = 0.011), with physical activity three or more times weekly associated with lower adjusted odds (aOR = 0.516; 95% CI: 0.324–0.823; *p* = 0.005); physical activity was not statistically significant at the ≥7-point threshold (overall *p* = 0.336). Some of the remaining estimates varied across thresholds. Full results are presented in Supplementary Table S7. In the expanded-covariate sensitivity analysis, eight participants remained unclassified after reconstruction of the constant-tinnitus variable. The model in which these responses remained unclassified included 1180 participants, and constant tinnitus remained associated with the highest proxy-score category (aOR = 2.567; 95% CI: 1.778–3.706; *p* < 0.001). In two extreme-case sensitivity analyses, the eight remaining unclassified responses were alternatively classified as no constant tinnitus and as constant tinnitus, yielding an analytical sample of 1188 participants in each model. The association remained significant when all eight responses were classified as no constant tinnitus (aOR = 2.534; 95% CI: 1.759–3.649; *p* < 0.001) and when all eight were classified as constant tinnitus (aOR = 2.830; 95% CI: 1.975–4.055; *p* < 0.001). Full results are presented in Supplementary Table S4. In an additional sensitivity analysis excluding margarine from the proxy-score construction, the six-item score was categorized using empirical cut-points of 0–3, 4–5, and ≥6 points, yielding 469, 424, and 392 participants, respectively. The repeated multivariable model included 1204 participants. The direction and magnitude of the principal associations were preserved. Constant tinnitus remained associated with the highest score category (aOR = 2.372; 95% CI: 1.648–3.415; *p* < 0.001), as did weekly and daily energy-drink consumption (aOR = 2.855; 95% CI: 2.010–4.057; *p* < 0.001 and aOR = 2.794; 95% CI: 1.414–5.521; *p* = 0.003, respectively). Lower academic achievement and both tired and variable subjective feelings after sleep also remained associated with the outcome. The association with alcohol consumption was attenuated and did not reach conventional statistical significance (aOR = 1.307; 95% CI: 0.996–1.715; *p* = 0.053). Physical activity was not associated with the outcome overall (*p* = 0.406). Complete results are presented in Supplementary Table S5. In a common complete-case sample of 1191 participants, the original 0–1–2 and approximate weekly equivalent score specifications produced broadly consistent findings. Among participants with complete data for all seven component items, the corresponding continuous scores were strongly correlated (Spearman’s ρ = 0.883, *p* < 0.001; n = 1008). Female sex was associated with lower adjusted odds under both specifications. Lower academic achievement, feeling tired after sleep, weekly and daily energy-drink consumption, and constant tinnitus remained associated with the highest score category in both models. Alcohol consumption was associated with the outcome in the original 0–1–2 model but not in the weekly equivalent model. Conversely, physical activity was associated with the outcome only under the weekly equivalent specification (overall *p* = 0.005), with physical activity three or more times weekly associated with lower adjusted odds (aOR = 0.627; 95% CI: 0.399–0.985; *p* = 0.043). Full side-by-side results are presented in Supplementary Table S8. An ordinal logistic regression model retaining all three proxy-score categories and including the same covariates as the final primary model was statistically significant overall (likelihood-ratio χ^2^(13) = 163.792, *p* < 0.001; Nagelkerke’s R^2^ = 0.143; n = 1204). The test of parallel lines was non-significant (χ^2^(13) = 19.459, *p* = 0.110), indicating no evidence that the proportional-odds assumption was violated. The Pearson and deviance goodness-of-fit tests were also non-significant (χ^2^(877) = 838.287, *p* = 0.822 and χ^2^(877) = 895.725, *p* = 0.323, respectively). Full results are presented in Supplementary Table S9. Multicollinearity was not considered problematic (tolerance range: 0.423–0.982; VIF range: 1.018–2.365).

In the sensitivity model additionally adjusted for grouped age and maternal and paternal educational levels (n = 1180), the direction and magnitude of the principal associations remained materially unchanged. Age was not associated with the outcome overall (*p* = 0.747), nor were maternal education (aOR = 1.080, 95% CI: 0.810–1.439; *p* = 0.601) or paternal education (aOR = 1.195, 95% CI: 0.897–1.591; *p* = 0.224). Physical activity also remained non-significant overall (*p* = 0.133). The expanded model was statistically significant (likelihood-ratio χ^2^(18) = 140.180, *p* < 0.001; Nagelkerke’s R^2^ = 0.153), and the Hosmer–Lemeshow test was non-significant (χ^2^(8) = 8.184, *p* = 0.416). Full results are presented in Supplementary Table S10.

## 4. Discussion

In this cross-sectional study, being in the highest category of the frequency-based proxy UPF score co-occurred with a broader pattern of academic, sleep-related, behavioral, and health characteristics among adolescents. In the multivariable model, female sex was associated with lower odds of being in the highest category; whereas, lower academic achievement, alcohol consumption, more frequent energy-drink consumption, and constant tinnitus were associated with higher odds. The overall associations with reported sleep duration, subjective feeling after a night’s sleep, and physical activity were not statistically significant, although waking tired was associated with higher odds than waking rested in the category-specific contrast. These findings are exploratory and hypothesis-generating and do not establish temporal direction or causality. The strongest associations were observed for energy drink consumption and constant tinnitus, supporting further investigation using validated dietary measures and longitudinal designs. The findings are broadly consistent with previous literature linking greater UPF exposure with adverse sleep- and mental-health-related outcomes, although associations vary across populations, outcomes, and assessment methods [6,16].

Female participants had lower adjusted odds of being in the highest proxy-score category than male participants. This finding is broadly compatible with the lower absolute UPF consumption by weight reported among female adolescents by Chavez-Ugalde et al., although that study found no clear sex difference when UPF consumption was expressed as a proportion of total energy intake [17]. More broadly, the sociodemographic patterning of UPF consumption appears to vary substantially across populations. In their systematic review of nationally representative studies, Dicken et al. found that associations with education, income, and socioeconomic position differed according to country and food-system context [18]. In the present sample, lower maternal and paternal educational attainment was more common in the higher proxy-score categories, suggesting that family socioeconomic circumstances may contribute to the observed dietary pattern. However, rather than indicating a universal social gradient, these findings may reflect the specific availability, affordability, marketing, and social meaning of UPF-related foods within the local adolescent food environment. Further studies using multidimensional indicators of household socioeconomic position are needed to clarify how family resources and food environments shape UPF-related dietary behavior among adolescents in Serbia.

Lower academic achievement was associated with higher odds of being in the highest proxy-score category after adjustment for the measured covariates, with the association concentrated among adolescents in the lowest achievement category. Direct evidence specifically linking UPF consumption with academic outcomes during adolescence remains limited. López-Gil et al. reported progressively lower overall and subject-specific grades across increasing levels of UPF consumption among Spanish adolescents [7], while Nyaradi et al. found that greater adherence to a Western dietary pattern was associated with poorer mathematics and reading performance in Australian adolescents [19]. The direction of the present findings is consistent with both studies, although the pattern was not uniformly graded across all levels of academic achievement. Adolescents with higher, but not outstanding, achievement did not differ clearly from those with outstanding achievement; whereas, those in the lower achievement category had substantially higher odds of being in the highest proxy-score category. The concentration of the association in the lowest achievement category may reflect differences in dietary assessment, grading systems, or shared contextual factors. Given the cross-sectional design, the temporal ordering of dietary behavior and academic difficulties cannot be determined. The present study extends the limited evidence to Serbian adolescents; longitudinal studies using validated NOVA-based dietary assessment and record-based academic outcomes are needed to clarify temporal direction and shared contextual determinants [7].

The sleep-related findings suggest that the category-specific contrast for waking tired, rather than reported sleep duration, was the clearest sleep-related finding. Adolescents who reported waking tired had higher adjusted odds of being in the highest proxy-score category than those who reported waking rested; the estimate for a variable feeling after sleep was borderline and did not reach conventional statistical significance. This distinction is noteworthy because reported sleep duration and subjective experience upon waking capture different aspects of sleep and need not show the same pattern of association. The finding is broadly consistent with previous literature linking greater UPF exposure with less favorable sleep-related outcomes among young people [6]. Several non-mutually exclusive explanations remain possible: fatigue may influence food and beverage choices, dietary patterns may be associated with a less favorable feeling upon waking, or both may reflect shared behavioral, social, or daily routine factors. Given the cross-sectional design, the temporal direction of these associations cannot be determined [6].

Energy-drink consumption showed one of the strongest associations with the highest category of the frequency-based proxy UPF score after adjustment for the measured covariates, with both weekly and daily consumers having more than twice the adjusted odds of being in that category compared with adolescents who consumed energy drinks monthly or less often. Although energy drinks were not included in the construction of the proxy score, the two measures may not be conceptually independent, as frequent energy-drink consumption may form part of a broader consumer pattern characterized by greater reliance on commercially manufactured and packaged food and beverage products. Accordingly, although the association cannot be attributed to mathematical overlap in score construction, residual conceptual overlap remains: energy-drink consumption may capture part of the same broader pattern of commercially manufactured and packaged product consumption represented by the proxy score. Its adjusted association should therefore not be interpreted as fully independent of that broader dietary pattern. The absence of a further increase in the estimate among daily consumers does not support a clear dose–response gradient and instead suggests that frequent consumption, irrespective of whether it is weekly or daily, may identify a broader pattern of less favorable dietary behavior. This interpretation is supported by Wadolowska et al., who identified a Fast-food-Sedentary pattern among Polish teenagers characterized by the joint consumption of energy drinks, fast food, sweetened beverages, and sweets, together with meal skipping and prolonged screen time [20]. Similarly, Lebacq et al. reported that frequent energy drink consumption among Belgian adolescents was associated in both sexes with daily soft-drink consumption, weekly alcohol use, high screen exposure, and later bedtimes [21]. Taken together, these findings suggest that energy drink use is embedded within a wider constellation of convenience-oriented dietary choices and less structured daily routines rather than representing an isolated beverage preference. Shared social occasions, peer influences, commercial exposure, and easy access to packaged foods and stimulant beverages may contribute to this clustering. The present study extends this evidence by demonstrating an association between energy-drink consumption and the frequency-based proxy UPF score among Serbian adolescents. Longitudinal studies using validated dietary measures are needed to establish how these behaviors develop and reinforce one another over time.

Alcohol consumption was associated with higher odds of being in the highest proxy-score category after adjustment for the measured covariates. This finding is consistent with a nationwide Brazilian study of more than 95,000 adolescents, in which those with the highest UPF consumption were more than twice as likely to report frequent alcohol use as those with the lowest consumption [8]. The concordant findings across distinct adolescent populations support the interpretation that alcohol use and UPF-related dietary behavior frequently co-occur. A systematic review of qualitative research further identified several socio-ecological influences shared by adolescent alcohol use and unhealthy eating, including peer norms, pleasure-seeking, identity formation, and the use of food or alcohol in response to emotional difficulties [22]. These overlapping influences provide a plausible framework for the present association: alcohol use and UPF-related food choices may become embedded within the same social occasions, peer cultures, daily routines, and commercially shaped environments, rather than reflecting a direct effect of one behavior on the other. From a public health perspective, their co-occurrence suggests that adolescent dietary behavior and alcohol use may be better understood as interconnected components of a broader behavioral and social context than as isolated exposures. Nevertheless, the temporal ordering of these behaviors and the extent to which they share common determinants require clarification in longitudinal studies.

Constant tinnitus showed one of the strongest associations with the highest category of the frequency-based proxy UPF score after adjustment for the measured covariates, with adolescents reporting constant tinnitus having approximately 2.5 times the adjusted odds of being in that category. Direct evidence specifically relating UPF consumption to tinnitus remains scarce. The most directly relevant context is provided by our previous analysis of the same underlying school-based population, in which fresh fruit and vegetable consumption was inversely associated with tinnitus frequency; whereas, sweetened carbonated beverages, fast food, and white bread were associated with higher odds of tinnitus [13]. The present analysis extends that work rather than independently replicating it by shifting the focus from individual food groups to a composite frequency-based proxy UPF score. Evidence from other populations provides additional, although not directly comparable, support for examining tinnitus in relation to broader dietary characteristics. Zhang et al., in a recent systematic review and meta-analysis of observational studies in adults, reported that higher consumption of fruit, dietary fiber, dairy products, and caffeine was associated with a lower occurrence of tinnitus [23]. In a prospective study of older adults, Tang et al. similarly found that lower fruit- and cereal-fiber intake was associated with a higher incidence of tinnitus over ten years [24]. Neither study examined UPF consumption or adolescent populations, and their findings therefore cannot be directly equated with the present association. Several non-mutually exclusive candidate pathways warrant prospective testing. First, tinnitus and sleep disturbance may reinforce one another: tinnitus may interfere with sleep; whereas, poor sleep and hyperarousal may increase the salience or distress of tinnitus [25]. Second, frequent energy-drink use may mark stimulant exposure and irregular sleep routines that contribute to autonomic arousal; however, the present cross-sectional data cannot establish mediation or a direct effect of caffeine, particularly because the model already adjusted for energy-drink frequency and the measured sleep variables [9,10,25]. Third, shared adolescent environments may jointly promote leisure-noise exposure—through loud music, nightlife, or unsafe headphone use—and consumption of packaged foods and stimulant beverages, thereby producing correlated auditory and dietary patterns without a direct causal pathway between them [26]. Longitudinal studies combining repeated dietary and caffeine assessment with validated sleep measures, personal noise-exposure assessment, and objective audiological evaluation are needed to test temporal ordering and mediation. Taken together, the adult dietary findings and our previous adolescent results provide an epidemiological context suggesting that tinnitus may be related to broader dietary patterns and support further investigation using longitudinal designs, validated NOVA-based dietary assessment, and objective audiological evaluation.

The degree of industrial processing and nutritional quality are related but distinct dimensions, and foods classified as UPFs are not nutritionally homogeneous. The seven categories selected for the present proxy score largely represented products that are often energy-dense and may have less favorable nutritional profiles; however, the questionnaire did not measure nutrient composition, and the score cannot distinguish associations related to processing from those related to added sugars, salt, saturated fat, energy density, or low fiber and micronutrient content. Consistent with this distinction, a multinational prospective cohort study reported heterogeneous associations across UPF subgroups: artificially and sugar-sweetened beverages, animal-based products, and sauces, spreads, and condiments were associated with higher multimorbidity risk; whereas, ultra-processed breads and cereals and plant-based alternatives were not [27]. The pastries and baked goods component in the present score was especially heterogeneous, encompassing croissants, savory pastries, burek, puff-pastry products, and similar items, and should therefore be interpreted as a broad consumption indicator rather than as a nutritionally uniform exposure.

The public-health relevance of these findings lies primarily in the observed co-occurrence of the highest proxy-score category with academic, sleep-related, behavioral, and health characteristics. However, neither the proxy score nor the associated characteristics should be regarded as validated screening or intervention targets on the basis of the present cross-sectional findings. In particular, the association with constant tinnitus should guide further longitudinal research incorporating validated dietary assessment and objective audiological evaluation. Such studies are needed to clarify temporal relationships and determine whether the observed characteristics prospectively predict dietary or health outcomes.

### Strengths and Limitations

The principal strengths of this study include its large school-based sample selected using stratified multistage cluster probability sampling, the integrated assessment of dietary, academic, sleep-related, behavioral, and health characteristics, and the standardized assessment of tinnitus. Energy-drink consumption was analyzed separately from the proxy score, and the robustness of the main findings was examined through several sensitivity analyses. By extending our previous item-level analysis in the same underlying population [13] to a composite frequency-based proxy score, the present study provides a broader account of UPF-related dietary behavior among adolescents in an underrepresented Southeast European setting. Several limitations should nevertheless be considered. The cross-sectional design and reliance on predominantly self-reported data preclude conclusions regarding temporal direction or causality. Sleep was not assessed using validated objective measures, and tinnitus was not confirmed audiologically. The study-specific proxy score, derived from seven FFQ items, was not validated against a NOVA-based dietary assessment and should therefore be interpreted as a relative indicator within this sample rather than as a measure of absolute UPF intake. The proxy score also did not permit the contributions of industrial processing and nutritional composition to be distinguished, and the pastries and baked goods component represented a particularly heterogeneous product category. Although energy-drink consumption was excluded from score construction, it remains conceptually adjacent to the outcome because both measures may capture aspects of the same broader pattern of commercially manufactured and packaged product consumption. Consequently, the energy-drink association should not be interpreted as fully independent of the dietary behavior represented by the proxy score. Anthropometric measurements were planned for a subsequent phase of school-based fieldwork but could not be completed because the COVID-19-related school closures terminated in-person data collection. Consequently, BMI and weight status were unavailable for adjustment. Total dietary energy intake could not be estimated because the questionnaire did not collect sufficiently detailed information on portion sizes or quantities consumed. Residual confounding by adiposity and overall energy intake therefore cannot be excluded. Because fieldwork was interrupted before all 17 selected schools could be included, the achieved sample may not be fully representative of the target population of students attending four-year secondary schools in Belgrade. Participant-level data were unavailable for the five schools not reached before the interruption, precluding direct comparison of student profiles. Accordingly, the reported prevalence estimates should be interpreted as descriptive of the achieved sample rather than as precise population estimates; whereas, the reported associations reflect relationships observed within the analyzed sample. The complete-case analysis additionally excluded 81 participants (6.3%); although differences between included and excluded participants were small, selection related to missing covariate data cannot be excluded. The reason why the eight remaining unclassified constant-tinnitus observations were concentrated in the highest proxy-score category could not be determined from the available data. Informative missingness therefore cannot be excluded; however, the direction and statistical significance of the association were preserved in extreme-case sensitivity analyses in which all eight participants were alternatively classified as not having or as having constant tinnitus. No formal correction for multiple comparisons was applied across the descriptive, regression, and sensitivity analyses. Accordingly, the reported *p*-values should be interpreted cautiously as exploratory rather than confirmatory, and some nominally significant findings may reflect chance. Because school identifiers were unavailable in the de-identified analytical dataset, within-school clustering could not be modeled; standard errors may therefore be underestimated and confidence intervals somewhat too narrow. Residual confounding by other unmeasured factors also remains possible.

## 5. Conclusions

In this school-based sample of adolescents from Belgrade, lower academic achievement, waking tired rather than rested, alcohol consumption, frequent energy-drink consumption, and constant tinnitus were associated with higher odds of being in the highest category of the frequency-based proxy UPF score after adjustment for the measured covariates. The co-occurrence of these academic, sleep-related, behavioral, and health characteristics suggests that the proxy score may reflect a broader adolescent lifestyle pattern, with the strongest associations observed for energy-drink consumption and constant tinnitus. Because of the cross-sectional design and the study-specific proxy measure, these findings do not establish temporal direction or causality and should be confirmed in longitudinal studies using validated NOVA-based dietary assessment and objective audiological evaluation.

## Figures and Tables

**Table 1:** Characteristics of participants across categories of the frequency-based proxy UPF score.

	Lowest Category (n = 412)	Middle Category (n = 388)	Highest Category (n = 485)	*p*-Value
Sex, n (%)	n = 412	n = 388	n = 485	<0.001
Male	114 (27.7%)	156 (40.2%)	211 (43.5%)	
Female	298 (72.3%)	232 (59.8%)	274 (56.5%)	
School grade, n (%)	n = 412	n = 388	n = 485	0.253
First	89 (21.6%)	110 (28.4%)	110 (22.7%)	
Second	119 (28.9%)	97 (25.0%)	125 (25.8%)	
Third	75 (18.2%)	64 (16.5%)	100 (20.6%)	
Fourth	129 (31.3%)	117 (30.2%)	150 (30.9%)	
Academic achievement, n (%)	n = 407	n = 383	n = 483	<0.001
Lower academic achievement	74 (18.2%)	107 (27.9%)	188 (38.9%)	
Higher academic achievement	242 (59.5%)	217 (56.7%)	248 (51.3%)	
Outstanding academic achievement	91 (22.4%)	59 (15.4%)	47 (9.7%)	
Mother’s education, n (%)	n = 409	n = 384	n = 476	0.003
Lower education	153 (37.4%)	177 (46.1%)	231 (48.5%)	
Higher education	256 (62.6%)	207 (53.9%)	245 (51.5%)	
Father’s education, n (%)	n = 408	n = 383	n = 482	0.003
Lower education	173 (42.4%)	193 (50.4%)	259 (53.7%)	
Higher education	235 (57.6%)	190 (49.6%)	223 (46.3%)	
Sleep duration, n (%)	n = 411	n = 388	n = 482	0.513
≤6 h	163 (39.7%)	132 (34.0%)	178 (36.9%)	
Approximately 7 h	129 (31.4%)	141 (36.3%)	160 (33.2%)	
≥8 h	119 (29.0%)	115 (29.6%)	144 (29.9%)	
Subjective feeling after night’s sleep, n (%)	n = 411	n = 384	n = 481	0.015
Tired	108 (26.3%)	115 (29.9%)	167 (34.7%)	
Variable	229 (55.7%)	195 (50.8%)	252 (52.4%)	
Rested	74 (18.0%)	74 (19.3%)	62 (12.9%)	
Alcohol consumption, n (%)	n = 397	n = 375	n = 476	<0.001
No	205 (51.6%)	167 (44.5%)	165 (34.7%)	
Yes	192 (48.4%)	208 (55.5%)	311 (65.3%)	
Smoking, n (%)	n = 406	n = 386	n = 480	<0.001
No	356 (87.7%)	319 (82.6%)	366 (76.3%)	
Yes	50 (12.3%)	67 (17.4%)	114 (23.8%)	
Energy drink consumption frequency, n (%)	n = 400	n = 386	n = 481	<0.001
Monthly or less often	371 (92.8%)	334 (86.5%)	345 (71.7%)	
Weekly	21 (5.3%)	44 (11.4%)	108 (22.5%)	
Daily	8 (2.0%)	8 (2.1%)	28 (5.8%)	
Physical activity, n (%)	n = 412	n = 388	n = 485	0.026
None	27 (6.6%)	36 (9.3%)	52 (10.7%)	
Once or twice weekly	86 (20.9%)	92 (23.7%)	130 (26.8%)	
Three or more times weekly	299 (72.6%)	260 (67.0%)	303 (62.5%)	
Sedative medication use, n (%)	n = 404	n = 382	n = 473	0.055
No	381 (94.3%)	369 (96.6%)	439 (92.8%)	
Yes	23 (5.7%)	13 (3.4%)	34 (7.2%)	
Gambling participation, n (%)	n = 412	n = 388	n = 485	<0.001
No gambling participation	322 (78.2%)	271 (69.8%)	300 (61.9%)	
Chance-based games	32 (7.8%)	30 (7.7%)	46 (9.5%)	
Sports betting	38 (9.2%)	62 (16.0%)	99 (20.4%)	
Higher-risk gambling activities	20 (4.9%)	25 (6.4%)	40 (8.2%)	
Constant tinnitus, n/N (%)	41/412 (10.0%)	29/388 (7.5%)	95/477 (19.9%)	<0.001
No	371 (90.0%)	359 (92.5%)	382 (80.1%)	
Yes	41 (10.0%)	29 (7.5%)	95 (19.9%)	

Note: Percentages were calculated using available-case denominators for each variable. For constant tinnitus, valid denominators within each proxy-score category are shown as n/N because of item-level missingness.

**Table 2:** Univariable associations with the highest category of the frequency-based proxy UPF score.

Variable	Crude OR (95% CI)	*p*-Value
Sex, female vs. male	0.662 (0.525–0.834)	<0.001
Academic achievement		<0.001
Lower vs. outstanding	3.315 (2.253–4.877)	<0.001
Higher vs. outstanding	1.724 (1.200–2.477)	0.003
Sleep duration (hours)		0.967
≤6 h vs. ≥8 h	0.981 (0.742–1.296)	0.890
Approximately 7 h vs. ≥8 h	0.963 (0.724–1.281)	0.795
Subjective feeling after a night’s sleep		0.006
Tired vs. rested	1.788 (1.250–2.557)	0.001
Variable vs. rested	1.419 (1.015–1.983)	0.041
Alcohol consumption, yes vs. no	1.753 (1.385–2.219)	<0.001
Smoking, yes vs. no	1.797 (1.348–2.396)	<0.001
Energy drink consumption frequency		<0.001
Weekly vs. monthly or less often	3.395 (2.432–4.740)	<0.001
Daily vs. monthly or less often	3.576 (1.909–6.698)	<0.001
Sedative medication use, yes vs. no	1.614 (0.995–2.616)	0.052
Gambling participation		<0.001
Chance-based games vs. no gambling	1.467 (0.977–2.201)	0.064
Sports betting vs. no gambling	1.957 (1.434–2.670)	<0.001
Higher-risk gambling activities vs. no gambling	1.757 (1.123–2.750)	0.014
Physical activity		0.020
Once or twice weekly vs. none	0.885 (0.575–1.362)	0.578
Three or more times weekly vs. none	0.657 (0.443–0.973)	0.036
Constant tinnitus, yes vs. no	2.792 (1.998–3.902)	<0.001

Note: The highest category of the frequency-based proxy UPF score was modeled as the outcome, with the combined lowest and middle categories as the reference group. OR, odds ratio; CI, confidence interval; UPF, ultra-processed food.

**Table 3:** Multivariable associations with the highest category of the frequency-based proxy UPF score.

Variable	Adjusted OR (95% CI)	*p*-Value
Sex, female vs. male	0.701 (0.538–0.914)	0.009
Academic achievement		0.001
Lower vs. outstanding	2.102 (1.378–3.207)	0.001
Higher vs. outstanding	1.331 (0.904–1.961)	0.147
Sleep duration		0.872
≤6 h vs. ≥8 h	1.085 (0.797–1.476)	0.604
Approximately 7 h vs. ≥8 h	1.037 (0.757–1.420)	0.820
Subjective feeling after a night’s sleep		0.075
Tired vs. rested	1.575 (1.055–2.349)	0.026
Variable vs. rested	1.440 (0.999–2.076)	0.051
Alcohol consumption, yes vs. no	1.344 (1.039–1.738)	0.024
Energy drink consumption frequency		<0.001
Weekly vs. monthly or less	2.770 (1.942–3.952)	<0.001
Daily vs. monthly or less	2.359 (1.180–4.715)	0.015
Constant tinnitus, yes vs. no	2.548 (1.768–3.672)	<0.001
Physical activity		0.136
Once or twice weekly vs. none	0.896 (0.554–1.448)	0.653
Three or more times weekly vs. none	0.706 (0.453–1.102)	0.126

Note: The highest category of the frequency-based proxy UPF score was modeled as the outcome, with the combined lowest and middle categories as the reference group. The model included sex, academic achievement, sleep duration, subjective feeling after a night’s sleep, alcohol consumption, energy-drink consumption frequency, constant tinnitus, and physical activity. The analysis included 1204 participants with complete data for all variables included in the model. CI, confidence interval; UPF, ultra-processed food.

## Data Availability

The data supporting the findings of this study are available from the corresponding author upon reasonable request.

## Supplementary Materials

The following supporting information can be downloaded at: https://www.mdpi.com/article/10.3390/nu18162603/s1, Supplementary S1: Construction and scoring of the frequency-based proxy UPF score; Table S1: Construction and scoring of the frequency-based proxy UPF score; Supplementary S2: Sensitivity Analysis Using the Prorated Proxy Score; Table S2: Sensitivity Analysis Using the Prorated Proxy Score; Table S3: Comparison of the previous and present analyses based on the same underlying school-based adolescent study population; Table S4: Expanded-covariate sensitivity analyses of the association between constant tinnitus and the highest category of the frequency-based proxy UPF score under alternative classifications of the eight remaining unclassified tinnitus responses; Table S5: Sensitivity analysis of multivariable associations using the six-item frequency-based proxy UPF score excluding margarine; Table S6: Comparison of participants included in and excluded from the final primary complete-case multivariable model; Table S7: Sensitivity analyses of selected multivariable associations using alternative thresholds for the frequency-based proxy UPF score; Table S8: Comparison of multivariable associations for the original 0–1–2 frequency-based proxy UPF score and the approximate weekly equivalent score in a common complete-case sample; Table S9: Ordinal logistic regression analysis of the original three-category frequency-based proxy UPF score; Table S10: Multivariable logistic regression sensitivity analysis with additional adjustment for grouped age and maternal and paternal educational levels.
