# Supplementary material for: A Frequency-Based Proxy Score for Ultra-Processed Food Consumption Among Adolescents: Associations with Lifestyle Factors and Tinnitus
Source: Nutrients. 2026 Aug 10;18(16):2603. doi: 10.3390/nu18162603 (PMC13516126; doi:10.3390/nu18162603)
Supplement: Supplementary file 1 [file nutrients-18-02603-s001.zip › nutrients-4452098-supplementary.pdf]

Supplementary Materials

Supplementary Methods

S1. Construction and Scoring of the Frequency-Based Proxy UPF Score

The original food frequency questionnaire (FFQ) was not specifically designed to quantify ultra-processed food intake according to the NOVA classification. Therefore, seven questionnaire items were selected as indicators of the reported frequency of consumption of food categories commonly represented in the Serbian market by commercially prepared, packaged, or industrially formulated products. Most selected categories were broadly consistent with NOVA group 4; however, classification may vary according to product formulation, particularly for margarine, which may fall within NOVA group 2 or group 4 [1]. The selection was informed by a review of representative product labels available on the Serbian market. Because the questionnaire did not capture the specific brand, ingredient composition, or preparation method of the product consumed by each participant, the resulting measure was retained as a frequency-based proxy UPF score rather than interpreted as a definitive individual-level NOVA classification. The selected items comprised fast food, commercial sauces and condiments, bakery pastries and puff-pastry products, margarine, packaged snacks, sugar-sweetened carbonated beverages, and French fries.

For each selected item, consumption frequency was coded as 0 for monthly or less frequent consumption, 1 for weekly consumption, and 2 for daily or more frequent consumption. The primary frequency-based proxy UPF score was calculated as the sum of the available component-item scores, with higher values indicating more frequent consumption of the selected food categories. The theoretical full-score range was 0–14, and the observed range in the study sample was 0–12.

Based on the empirical distribution of the total score, participants were grouped into three categories using empirical tertile cut-points: the lowest category (0–3 points), the middle category (4–5 points), and the highest category (≥6 points). Unequal group sizes reflected tied values in the discrete score distribution. For binary logistic regression analyses, the highest score category was modeled as the outcome, with the combined lowest and middle categories as the reference group.

The score was intended as an exploratory, frequency-based indicator of relative exposure to the selected UPF-related food categories within this study population. It was not designed or validated to quantify absolute NOVA-defined UPF intake, portion size, or the proportion of dietary energy derived from UPFs. Energy-drink consumption was not included in the construction of the proxy score and was analyzed separately as a lifestyle variable.

To assess robustness to incomplete component responses, a sensitivity analysis was conducted using a prorated score among participants with at least six valid responses across the seven component items. The prorated score was calculated as the mean of the available item scores multiplied by seven and was categorized using the same cut-points as the primary score. The corresponding dichotomized outcome was then used to repeat the multivariable logistic regression model.

Supplementary Table S1. Construction and scoring of the frequency-based proxy UPF score

No.	FFQ item included in the proxy score	Examples specified in the questionnaire	Rationale for inclusion in the proxy score	Response coding
1	Fast food consumption	Hamburgers, pizza, chicken nuggets, and similar products	Proxy indicator of consumption of commercially prepared and industrially formulated fast-food products in the study context	0 = monthly or less often; 1 = weekly; 2 = daily or more often
2	Commercial sauces and condiments consumed with meals	Ketchup and mayonnaise	Proxy indicator of consumption of commercially manufactured packaged sauces and condiments	0 = monthly or less often; 1 = weekly; 2 = daily or more often
3	Bakery pastries and puff-pastry products	Croissants, savory pastries, burek, puff-pastry products, and similar items	Proxy indicator of consumption of commercially prepared bakery and pastry products	0 = monthly or less often; 1 = weekly; 2 = daily or more often
4	Margarine consumption	Margarine	Potential proxy indicator of an industrially manufactured fat spread; NOVA classification may vary according to product formulation.	0 = monthly or less often; 1 = weekly; 2 = daily or more often
5	Packaged snack consumption	Packaged snacks	Proxy indicator of packaged industrial snack consumption	0 = monthly or less often; 1 = weekly; 2 = daily or more often
6	Sugar-sweetened carbonated beverage consumption	Sugar-sweetened carbonated beverages	Proxy indicator of commercially manufactured sugar-sweetened soft-drink consumption	0 = monthly or less often; 1 = weekly; 2 = daily or more often
7	French fries consumption	French fries	Proxy indicator of commercially prepared fried fast-food consumption	0 = monthly or less often; 1 = weekly; 2 = daily

Note: The primary frequency-based proxy UPF score was calculated as the sum of the available component-item scores. The theoretical full-score range was 0–14, and the observed range was 0–12. Participants were categorized as follows: lowest category, 0–3 points (n = 412; 32.1%); middle category, 4–5 points (n = 388; 30.2%); and highest category, ≥6 points (n = 485; 37.7%). Unequal group sizes reflected tied values in the discrete score distribution. Energy-drink consumption was excluded from the proxy score and analyzed separately. FFQ, food frequency questionnaire; UPF, ultra-processed food.

Supplementary Results

S2. Sensitivity Analysis Using the Prorated Proxy Score

Among the 1264 participants with at least six valid responses across the seven component items, classification into the lowest, middle, and highest proxy-score categories based on the prorated score was identical to the primary classification for all eligible participants (100% concordance). The repeated multivariable model included 1191 participants and produced estimates broadly similar in direction and magnitude to those of the primary analysis. Female sex was associated with lower adjusted odds of being in the highest score category, whereas lower academic achievement, waking tired, alcohol consumption, weekly and daily energy-drink consumption, and constant tinnitus remained associated with higher adjusted odds. The overall association with subjective feeling after sleep was not statistically significant, although waking tired remained significant compared with waking rested. Physical activity was not associated with the outcome overall.

Supplementary Table S2. Sensitivity Analysis Using the Prorated Proxy Score

Variable	Adjusted OR (95% CI)	p-value
**Sex, female vs. male**	0.692 (0.530–0.902)	**0.007**
**Academic achievement**	—	**<0.001**
Lower vs. outstanding	2.115 (1.385–3.231)	<0.001
Higher vs. outstanding	1.322 (0.897–1.947)	0.158
**Sleep duration**	—	**0.868**
≤6 h vs. ≥8 h	1.087 (0.798–1.481)	0.598
Approximately 7 h vs. ≥8 h	1.038 (0.757–1.422)	0.818
**Subjective feeling after a night's sleep**	—	**0.104**
Tired vs. rested	1.537 (1.029–2.295)	0.036
Variable vs. rested	1.395 (0.967–2.013)	0.075
**Alcohol consumption, yes vs. no**	1.332 (1.029–1.723)	**0.029**
**Energy-drink consumption frequency**	—	**<0.001**
Weekly vs. monthly or less often	2.653 (1.856–3.793)	<0.001
Daily vs. monthly or less often	2.318 (1.159–4.636)	0.017
**Constant tinnitus, yes vs. no**	2.553 (1.768–3.686)	**<0.001**
**Physical activity**	—	**0.138**
Once or twice weekly vs. none	0.913 (0.564–1.477)	0.710
Three or more times weekly vs. none	0.715 (0.458–1.117)	0.140

Note: The prorated frequency-based proxy UPF score was calculated among participants with at least six valid responses across the seven component items as the mean of the available item scores multiplied by seven. The score was categorized using the same cut-off values as the primary score. The highest category was modeled as the outcome, with the lowest and middle categories combined as the reference group. The prorated score was available for 1264 participants, and the complete-case multivariable analysis included 1191 participants. The model included sex, academic achievement, sleep duration, subjective feeling after a night's sleep, alcohol consumption, energy-drink consumption frequency, constant tinnitus, and physical activity. *The category following "vs." was the reference category. Model fit was acceptable (likelihood-ratio $\chi^2(13) = 132.552$, $p < 0.001$; Nagelkerke's $R^2 = 0.143$; Hosmer–Lemeshow $\chi^2(8) = 6.794$, $p = 0.559$). aOR, adjusted odds ratio; CI, confidence interval; UPF, ultra-processed food.

Supplementary Table S3. Comparison of the previous and present analyses based on the same underlying school-based adolescent study population.

Characteristic	Previous publication [13]	Present study
**Data source and study population**	Data from the same original school-based survey of adolescents attending 12 four-year secondary schools in Belgrade, Serbia	Data from the same original school-based survey and target population
**Analytic sample and participant overlap**	The analytic sample included participants with the data required for the individual dietary-factor and tinnitus analyses	The overall sample included 1285 participants; analysis-specific sample sizes varied according to missing data, and 1204 participants were included in the final primary complete-case multivariable model
**Primary research question**	Whether individual dietary factors were associated with tinnitus frequency	Which sociodemographic, academic, sleep-related, behavioral, and health-related factors were associated with the highest category of the frequency-based proxy UPF score
**Dietary variables**	Individual food groups and items, including fresh fruit and vegetables, sweetened carbonated beverages, fast food, and white bread	A composite frequency-based proxy UPF score constructed from seven selected food categories; energy-drink consumption was analyzed separately
**Role of tinnitus**	Tinnitus frequency was the principal outcome	Constant tinnitus was examined as a health-related correlate of the highest proxy-score category
**Analytical approach**	Associations between individual dietary variables and tinnitus were examined separately	Sensitivity analyses examined a prorated score, alternative score thresholds, exclusion of margarine, an ordinal outcome model, extreme-case classifications of missing constant-tinnitus responses, and additional adjustment for age and parental education.
**Incremental contribution**	Identification of associations between individual dietary factors and tinnitus	Evaluation of whether selected food-frequency indicators, considered jointly as a composite proxy measure, co-occurred with a broader pattern of academic, sleep-related, behavioral, and health-related characteristics
**Overall analytical dataset**	The earlier publication reported an overall sample of 1287 participants.	For the present study, additional data cleaning and consistency checks yielded a final analytical dataset of 1285 participants.
**Constant-tinnitus completeness and classification**	Completeness was assessed at the level of the full six-item Tinnitus Screener, yielding 1003 complete questionnaires, 284 incomplete questionnaires, and 134 constant-tinnitus cases.	Constant tinnitus was reconstructed from the original item-level responses. Of 31 initially unclassified responses to the classification item, 23 were classified as no constant tinnitus following review of the remaining items, leaving eight unclassified. The final variable comprised 1277 valid classifications, including 165 constant-tinnitus cases.

Note: Both studies were based on the same original school-based survey. Analytic samples differed according to the variables required for each analysis and the corresponding missing-data criteria. UPF, ultra-processed food. The same Tinnitus Screener and substantive definition of constant tinnitus were used in both analyses; the analyses differed in the completeness criterion applied when deriving the analytical variable.

Supplementary Table S4. Expanded-covariate sensitivity analyses of the association between constant tinnitus and the highest category of the frequency-based proxy UPF score under alternative classifications of the eight remaining unclassified tinnitus responses.

Missing-response classification	Analytical sample, n	aOR (95% CI)	p-value
Reconstructed classification, with eight participants retained as unclassified	1180	2.567 (1.778–3.706)	<0.001
All eight remaining unclassified responses assigned no constant tinnitus	1188	2.534 (1.759–3.649)	<0.001
All eight remaining unclassified responses assigned constant tinnitus	1188	2.830 (1.975–4.055)	<0.001

Note: The highest category of the frequency-based proxy UPF score was modeled as the outcome, with the combined lowest and middle categories as the reference group. All models were adjusted for sex, academic achievement, sleep duration, subjective feeling after a night's sleep, alcohol consumption, energy-drink consumption frequency, physical activity, grouped age, maternal education, and paternal education. In the two extreme-case analyses, the eight participants who remained unclassified after reconstruction of the constant-tinnitus variable were alternatively classified as not having constant tinnitus and as having constant tinnitus. aOR, adjusted odds ratio; CI, confidence interval; UPF, ultra-processed food.

Supplementary Table S5. Sensitivity analysis of multivariable associations using the six-item frequency-based proxy UPF score excluding margarine (n = 1204).

Variable	aOR (95% CI)	p-value
**Sex**, female vs. male	0.839 (0.634–1.110)	0.219
**Academic achievement**	—	**0.005**
Lower vs. outstanding	1.874 (1.192–2.944)	0.006
Higher vs. outstanding	1.223 (0.804–1.860)	0.346
**Sleep duration**	—	**0.738**
≤6 h vs. ≥8 h	1.138 (0.819–1.580)	0.441
Approximately 7 h vs. ≥8 h	1.059 (0.756–1.483)	0.740
**Subjective feeling after a night's sleep**	—	**0.004**
Tired vs. rested	2.111 (1.359–3.278)	0.001
Variable vs. rested	1.670 (1.104–2.524)	0.015
**Alcohol consumption**, yes vs. no	1.307 (0.996–1.715)	0.053
**Energy-drink consumption frequency**	—	**<0.001**
Weekly vs. monthly or less often	2.855 (2.010–4.057)	<0.001
Daily vs. monthly or less often	2.794 (1.414–5.521)	0.003
**Constant tinnitus, yes vs. no**	2.372 (1.648–3.415)	<**0.001**
**Physical activity**	—	**0.406**
Once or twice weekly vs. none	0.821 (0.501–1.344)	0.432
Three or more times weekly vs. none	0.741 (0.470–1.168)	0.197

Note: In an additional sensitivity analysis excluding margarine from the proxy-score construction, the six-item score was categorized using empirical cut-points of 0–3, 4–5, and ≥6 points, yielding 469, 424, and 392 participants, respectively. The repeated multivariable model included 1204 participants. The principal associations with energy-drink consumption and constant tinnitus were preserved. Constant tinnitus remained associated with the highest score category (aOR = 2.372; 95% CI: 1.648–3.415; $p < 0.001$), as did weekly and daily energy-drink consumption (aOR = 2.855; 95% CI: 2.010–4.057; $p < 0.001$ and aOR = 2.794; 95% CI: 1.414–5.521; $p = 0.003$, respectively). Lower academic achievement and both tired and variable subjective feelings after sleep also remained associated with the outcome. The association with alcohol consumption was attenuated and did not reach conventional statistical significance (aOR = 1.307; 95% CI: 0.996–1.715; $p = 0.053$). Physical activity was not associated with the outcome overall ($p = 0.406$).

Supplementary Table S6. Comparison of participants included in and excluded from the final primary complete-case multivariable model (n = 1285).

Characteristic	Excluded from the final primary model, n (%)	Included in the final primary model, n (%)	Pearson χ^2 (df)	p-value	Cramer's V
Overall sample	81 (6.3)	1204 (93.7)	—	—	—
**Sex**	—	—	6.417 (1)	**0.011**	0.071
Male	41 (8.5)	440 (91.5)			
Female	40 (5.0)	764 (95.0)			
**Proxy UPF-score category**	—	—	0.273 (2)	**0.872**	0.015
Lowest	28 (6.8)	384 (93.2)			
Middle	23 (5.9)	365 (94.1)			
Highest	30 (6.2)	455 (93.8)			
**Academic achievement**[a]	—	—	1.164 (2)	**0.559**	0.030
Lower	23 (6.2)	346 (93.8)			
Higher	34 (4.8)	673 (95.2)			
Outstanding	12 (6.1)	185 (93.9)			

Note: Values are presented as n (%), with percentages calculated within each characteristic category. Model inclusion was defined according to the final primary complete-case multivariable model presented in Table 3. Pearson's chi-square test was used for comparisons, and Cramer's V is reported as the measure of effect size. [a] The academic-achievement comparison was restricted to participants with non-missing academic-achievement data (n = 1273). The remaining 12 participants had missing academic-achievement data and were therefore excluded from the final complete-case model. UPF, ultra-processed food.

Supplementary Table S7. Sensitivity analyses of selected multivariable associations using alternative thresholds for the frequency-based proxy UPF score.

Predictor	Alternative threshold ≥5, aOR (95% CI)	Primary threshold ≥6, aOR (95% CI)	Alternative threshold ≥7, aOR (95% CI)
**Academic achievement**	**p < 0.001**	**p = 0.001**	**p = 0.007**
Lower vs. outstanding	2.348 (1.582–3.485)	2.102 (1.378–3.207)	1.904 (1.147–3.160)
Higher vs. outstanding	1.574 (1.113–2.226)	1.331 (0.904–1.961)	1.221 (0.758–1.968)
**Sleep duration**	**p = 0.535**	**p = 0.872**	**p = 0.781**
≤6 h vs. ≥8 h	0.854 (0.634–1.151)	1.085 (0.797–1.476)	0.969 (0.682–1.376)
Approximately 7 h vs. ≥8 h	0.967 (0.713–1.310)	1.037 (0.757–1.420)	0.884 (0.616–1.268)
**Subjective feeling after a night's sleep**	**p = 0.094**	**p = 0.075**	**p = 0.065**
Tired vs. rested	1.510 (1.035–2.201)	1.575 (1.055–2.349)	1.767 (1.097–2.847)
Variable vs. rested	1.234 (0.880–1.730)	1.440 (0.999–2.076)	1.487 (0.951–2.327)
**Alcohol consumption**, yes vs. no	1.120 (0.876–1.432)	1.344 (1.039–1.738)	1.189 (0.880–1.607)
**Energy-drink consumption frequency**	**p < 0.001**	**p < 0.001**	**p < 0.001**
Weekly vs. monthly or less often	2.804 (1.881–4.180)	2.770 (1.942–3.952)	3.608 (2.511–5.184)
Daily vs. monthly or less often	2.457 (1.122–5.377)	2.359 (1.180–4.715)	3.557 (1.790–7.071)
**Constant tinnitus**, yes vs. no	1.947 (1.322–2.866)	2.548 (1.768–3.672)	2.954 (2.027–4.306)
**Physical activity**	**p = 0.011**	**p = 0.136**	**p = 0.336**
Once or twice weekly vs. none	0.657 (0.398–1.084)	0.896 (0.554–1.448)	0.924 (0.544–1.571)
Three or more times weekly vs. none	0.516 (0.324–0.823)	0.706 (0.453–1.102)	0.753 (0.460–1.232)

Note: The dependent variable was membership in the category defined using thresholds of ≥5, ≥6, and ≥7 points, respectively. The ≥6 threshold was used in the primary analysis. All models used the same complete-case sample (n = 1204) and were adjusted for sex, academic achievement, sleep duration, subjective feeling after a night's sleep, alcohol consumption, energy-drink consumption frequency, constant tinnitus, and physical activity. Sex was included as an adjustment covariate in all models but is not displayed in the table. Reference categories were outstanding academic achievement, sleep duration ≥8 h, feeling rested after sleep, no alcohol consumption, energy-drink consumption monthly or less often, no constant tinnitus, and no physical activity. Overall p-values are shown for predictors with more than two categories. Model diagnostics were as follows: threshold ≥5, likelihood-ratio $\chi^2(13) = 126.598$, $p < 0.001$, Nagelkerke's $R^2 = 0.133$, Hosmer–Lemeshow $\chi^2(8) = 7.761$, $p = 0.457$; threshold ≥6, likelihood-ratio $\chi^2(13) = 137.276$, $p < 0.001$, Nagelkerke's $R^2 = 0.147$, Hosmer–Lemeshow $\chi^2(8) = 6.171$, $p = 0.628$; and threshold ≥7, likelihood-ratio $\chi^2(13) = 141.027$, $p < 0.001$, Nagelkerke's $R^2 = 0.167$, Hosmer–Lemeshow $\chi^2(8) = 6.457$, $p = 0.596$. *The category following "vs." was the reference category. aOR, adjusted odds ratio; CI, confidence interval; UPF, ultra-processed food.

Supplementary Table S8. Comparison of multivariable associations for the original 0–1–2 frequency-based proxy UPF score and the approximate weekly-equivalent score in a common complete-case sample (n = 1191).

Predictor	Original 0–1–2 score		Approximate weekly-equivalent score	
	aOR (95% CI)	p-value	aOR (95% CI)	p-value
**Sex, female vs. male**	0.692 (0.530–0.902)	0.007	0.736 (0.558–0.971)	0.030
**Academic achievement**		**<0.001**		**0.002**
Lower vs. outstanding	2.115 (1.385–3.231)	0.001	2.104 (1.338–3.310)	0.001
Higher vs. outstanding	1.322 (0.897–1.947)	0.158	1.385 (0.910–2.107)	0.129
**Sleep duration**		**0.868**		**0.564**
≤6 h vs. ≥8 h	1.087 (0.798–1.481)	0.598	0.916 (0.666–1.260)	0.590
Approximately 7 h vs. ≥8 h	1.038 (0.757–1.422)	0.818	0.837 (0.604–1.160)	0.285
**Subjective feeling after a night's sleep**		**0.104**		**0.086**
Tired vs. rested	1.537 (1.029–2.295)	0.036	1.580 (1.039–2.404)	0.033
Variable vs. rested	1.395 (0.967–2.013)	0.075	1.268 (0.860–1.870)	0.231
**Alcohol consumption, yes vs. no**	1.332 (1.029–1.723)	0.029	1.075 (0.820–1.409)	0.600
**Energy-drink consumption frequency**		**<0.001**		**<0.001**
Weekly vs. monthly or less often	2.653 (1.856–3.793)	<0.001	2.554 (1.794–3.635)	<0.001
Daily vs. monthly or less often	2.318 (1.159–4.636)	0.017	2.546 (1.286–5.041)	0.007
Constant tinnitus, yes vs. no	2.553 (1.768–3.686)	<0.001	1.983 (1.375–2.858)	<0.001
**Physical activity**		**0.138**		**0.005**
Once or twice weekly vs. none	0.913 (0.564–1.477)	0.710	0.984 (0.607–1.597)	0.949
Three or more times weekly vs. none	0.715 (0.458–1.117)	0.140	0.627 (0.399–0.985)	0.043

Note: Both models were fitted in the same complete-case sample (n = 1191) and included sex, academic achievement, sleep duration, subjective feeling after a night's sleep, alcohol consumption, energy-drink consumption frequency, constant tinnitus, and physical activity. In each model, the highest score category was compared with the combined lower and middle categories. For the original score, item frequencies were coded 0–1–2; for the approximate weekly-equivalent score, values of 0.25, 1, and 7 were assigned to monthly or less, weekly, and daily or more frequent consumption, respectively, and the score was prorated for participants with at least six valid component items. Reference categories were male sex, outstanding academic achievement, sleep duration ≥8 h, feeling rested after a night's sleep, no alcohol consumption, energy-drink consumption monthly or less often, no constant tinnitus, and no physical activity. Overall p-values are shown for predictors with more than two categories. Model diagnostics were as follows: original 0–1–2 score, likelihood-ratio $\chi^2(13) = 132.552$, $p < 0.001$, Nagelkerke's $R^2 = 0.143$, Hosmer–Lemeshow $\chi^2(8) = 6.794$, $p = 0.559$; approximate weekly-equivalent score, likelihood-ratio $\chi^2(13) = 111.125$, $p < 0.001$, Nagelkerke's $R^2 = 0.126$, Hosmer–Lemeshow $\chi^2(8) = 6.233$, $p = 0.621$. *The category following "vs." was the reference category. aOR, adjusted odds ratio; CI, confidence interval; UPF, ultra-processed food.

Supplementary Table S9. Ordinal logistic regression analysis of the original three-category frequency-based proxy UPF score (n = 1204).

Predictor	Adjusted proportional OR (95% CI)	p-value
Sex, female vs. male	0.615 (0.488–0.776)	<0.001
**Academic achievement**	—	—
Lower vs. outstanding	2.275 (1.595–3.245)	<0.001
Higher vs. outstanding	1.398 (1.024–1.910)	0.035
**Sleep duration**	—	—
≤6 h vs. ≥8 h	1.027 (0.787–1.340)	0.843
Approximately 7 h vs. ≥8 h	1.070 (0.815–1.405)	0.623
**Subjective feeling after a night's sleep**	—	—
Tired vs. rested	1.455 (1.037–2.042)	0.030
Variable vs. rested	1.250 (0.921–1.696)	0.153
Alcohol consumption, yes vs. no	1.292 (1.036–1.611)	0.023
**Energy-drink consumption frequency**	—	—
Weekly vs. monthly or less often	2.779 (1.978–3.908)	<0.001
Daily vs. monthly or less often	2.100 (1.089–4.047)	0.027
Constant tinnitus, yes vs. no	2.138 (1.520–3.004)	<0.001
**Physical activity**	—	—
Once or twice weekly vs. none	0.945 (0.613–1.456)	0.796
Three or more times weekly vs. none	0.698 (0.467–1.042)	0.078

Note: The ordinal logistic regression model retained the three ordered categories of the frequency-based proxy UPF score and included sex, academic achievement, sleep duration, subjective feeling after a night's sleep, alcohol consumption, energy-drink consumption frequency, constant tinnitus, and physical activity. Reference categories were male sex, outstanding academic achievement, sleep duration of ≥8 h, feeling rested after a night's sleep, no alcohol consumption, energy-drink consumption monthly or less often, no constant tinnitus, and no physical activity. Because the model was fitted using indicator-coded predictors, p-values are shown for individual category contrasts; overall p-values for predictors with more than two categories are not reported. The model was statistically significant (likelihood-ratio $\chi^2(13) = 163.792$, $p < 0.001$; Nagelkerke's $R^2 = 0.143$). The proportional-odds assumption was satisfied (test of parallel lines: $\chi^2(13) = 19.459$, $p = 0.110$). Pearson and deviance goodness-of-fit tests were non-significant ($\chi^2(877) = 838.287$, $p = 0.822$ and $\chi^2(877) = 895.725$, $p = 0.323$, respectively). Threshold parameters are not displayed. The category following "vs." was the reference category. OR, proportional odds ratio; CI, confidence interval; UPF, ultra-processed food.

Supplementary Table S10. Multivariable logistic regression sensitivity analysis with additional adjustment for grouped age and maternal and paternal educational levels (n = 1180).

Predictor	Adjusted OR (95% CI)	p-value
**Sex, female vs. male**	0.700 (0.534–0.920)	0.010
**Academic achievement**	—	**0.001**
Lower vs. outstanding	2.146 (1.377–3.344)	0.001
Higher vs. outstanding	1.357 (0.912–2.019)	0.132
**Sleep duration**	—	**0.932**
≤6 h vs. ≥8 h	1.048 (0.767–1.431)	0.771
Approximately 7 h vs. ≥8 h	0.994 (0.723–1.366)	0.968
**Subjective feeling after a night's sleep**	—	**0.074**
Tired vs. rested	1.593 (1.061–2.392)	0.025
Variable vs. rested	1.438 (0.991–2.086)	0.056
**Alcohol consumption,** yes vs. no	1.438 (1.095–1.889)	0.009
**Energy-drink consumption frequency**	—	**<0.001**
Weekly vs. monthly or less often	2.655 (1.854–3.802)	<0.001
Daily vs. monthly or less often	2.127 (1.053–4.295)	0.035
**Constant tinnitus, yes vs. no**	2.567 (1.778–3.706)	**<0.001**
**Physical activity**	—	**0.133**
Once or twice weekly vs. none	0.950 (0.585–1.543)	0.837
Three or more times weekly vs. none	0.730 (0.465–1.145)	0.171
**Grouped age**	—	**0.747**
≤15 years vs. ≥18 years	1.139 (0.791–1.641)	0.485
16 years vs. ≥18 years	0.988 (0.703–1.389)	0.947
17 years vs. ≥18 years	0.909 (0.630–1.311)	0.609
**Maternal educational level,** lower vs. higher	1.080 (0.810–1.439)	**0.601**
**Paternal educational level,** lower vs. higher	1.195 (0.897–1.591)	**0.224**

Note: The highest category of the frequency-based proxy UPF score was modeled as the outcome, with the combined lowest and middle categories as the reference group. The model included sex, academic achievement, sleep duration, subjective feeling after a night's sleep, alcohol consumption, energy-drink consumption frequency, constant tinnitus, physical activity, grouped age, and maternal and paternal educational levels. Reference categories were male sex, outstanding academic achievement, sleep duration of ≥8 h, feeling rested after a night's sleep, no alcohol consumption, energy-drink consumption monthly or less often, no constant tinnitus, no physical activity, age ≥18 years, and higher maternal and paternal educational levels. Parental educational level was dichotomized as lower (primary or secondary school education) and higher (postsecondary education, including higher school, college, or university education). Overall p-values are shown for predictors with more than two categories. The expanded model was statistically significant (likelihood-ratio $\chi^2(18) = 140.180$, $p < 0.001$; Nagelkerke's $R^2 = 0.153$), and the Hosmer–Lemeshow test was non-significant ($\chi^2(8) = 8.184$, $p = 0.416$). The category following "vs." was the reference category. aOR, adjusted odds ratio; CI, confidence interval; UPF, ultra-processed food.
